# NH-1,2,3-triazoles as versatile building blocks in denitrogenative transformations

**DOI:** 10.1039/d3ra06045d

**Published:** 2023-11-27

**Authors:** Vladimir Motornov, Petr Beier

**Affiliations:** a Institute of Orgranic Chemistry and Biochemistry, Academy of Sciences Flemingovo nám. 2 160 00 Prague 6 Czech Republic cuprate51@gmail.com beier@uochb.cas.cz

## Abstract

The utilization of NH-1,2,3-triazoles as easily accessible building blocks in denitrogenative ring cleavage transformations with electrophiles to provide multifunctionalized nitrogen heterocycles and *N*-alkenyl compounds is reviewed. Leveraging the ready availability of NH-1,2,3-triazoles, these processes provide a convenient route to a range of pharmaceutically relevant heterocyclic cores and *N*-alkenyl compounds. The synthetic usefulness of *in situ* acylated NH-1,2,3-triazoles as viable alternatives to widely explored *N*-sulfonyl-1,2,3-triazoles in ring cleavage processes is highlighted.

## Abstract 2

The utilization of NH-1,2,3-triazoles as easily accessible building blocks in denitrogenative ring cleavage transformations with electrophiles to provide multifunctionalized nitrogen heterocycles and *N*-alkenyl compounds is reviewed.

## Graphical abstract

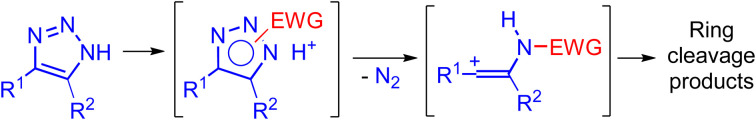

1,2,3-Triazoles are nitrogen heterocycles with versatile reactivity^1^ and great medicinal importance.^2^ Since the discovery of azide–alkyne click chemistry in 2002,^3^ triazole derivatives have gained enormous attention in organic, medicinal, biomolecular, and material sciences. Among them, 1,2,3-triazoles bearing an electron-withdrawing group at position N1 are of special importance because of their propensity to undergo N1–N2 bond cleavage in denitrogenative triazole ring opening transformations (Scheme 1a).^1^*N*-sulfonyl-1,2,3-triazoles^4^ and *N*-fluoroalkyl-1,2,3-triazoles^5^ are the most explored building blocks, which undergo ring cleavage under metal catalysis or by the action of Lewis or Brønsted acids. Very recently, a new strategy based on the use of NH-1,2,3-triazoles involving the installation of an electron-withdrawing group with *in situ* ring cleavage was described and used with success (Scheme 1b). The present review features the use of free NH-1,2,3-triazoles 1 in denitrogenative transformations, proceeding *via N*-acyl-1,2,3-triazoles or their analogues as key intermediates.

**Scheme 1:**
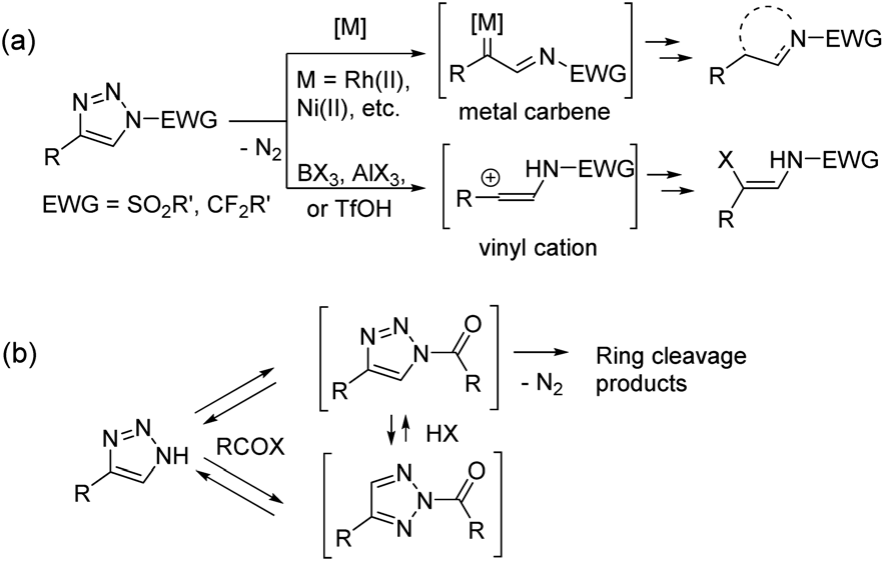
Schematic representation of the utilization of (a) *N*–sulfonyl- or *N*-fluoroalkyl-substituted 1,2,3-triazoles and (b) NH-1,2,3-triazoles in ring cleavage denitrogenative transformations.

*N*-unsubstituted NH-1,2,3-triazoles 1, considered in the present review, are the simplest and most readily available triazoles.^6^ They can be prepared by azide–alkyne cycloaddition^6^ or alternative methods such as cycloaddition/elimination with activated ketones^7^ or nitroalkenes.^7^ In the last five years, there has been a notable surge of innovative methods for the synthesis of NH-1,2,3-triazoles and several one-pot protocols from inexpensive and commercially available reagents have been developed.^6^ To underline the most efficient and practical routes, NH-1,2,3-triazoles were synthesized from TMSN_3_ and alkynes *via* CuI-catalysed cycloaddition (Scheme 2a),^8^ or sodium azide, aldehydes and nitroalkanes *via* a tandem Henry reaction/[3 + 2] cycloaddition (Scheme 2b),^9*a*–*c*^ including recently developed green chemistry approaches.^9*d*–*h*^ In 2022, NH-1,2,3-triazoles became available from NaN_3_/H_2_SO_4_ and alkynes, which is so far the simplest and the most straightforward route, although the generation of HN_3_ raises safety concerns (Scheme 2c).^10^ Finally, the approach utilizing azidyl radical–alkyne cycloaddition with the use of the NaN_3_/PhI(OAc)_2_ system in mild conditions is highly efficient for complex disubstituted triazoles such as 4,5-diaryltriazoles (Scheme 2d),^11*a*^ and it was also possible to efficiently synthesize these compounds without an oxidant, albeit only under harsh conditions (MW heating at 200 °C).^11*b*^ More examples of novel synthetic methods to access NH-1,2,3-triazoles appeared in recent reviews.^6^

**Scheme 2:**
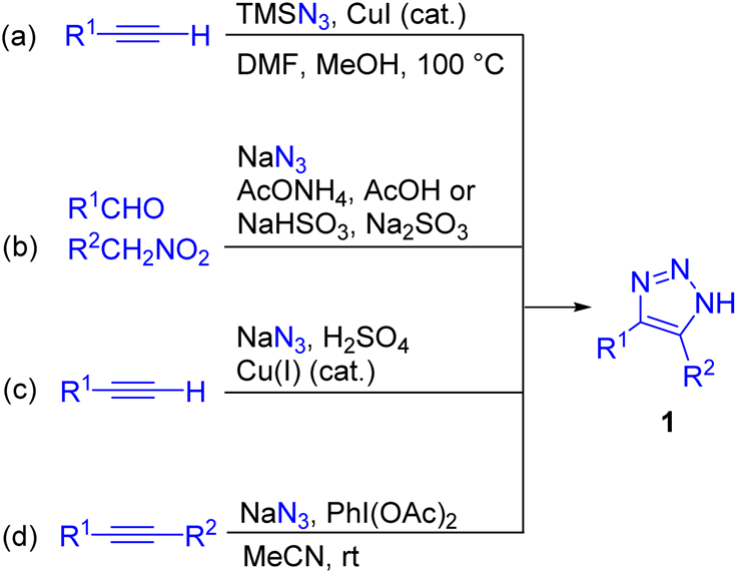
Overview of efficient routes for the synthesis of NH-1,2,3-triazoles (a–d).

Due to better atom economy, the utilization of readily available NH-triazoles is advantageous compared to the use of *N*-sulfonyl- or *N*-fluoroalkyl-triazoles. Additionally, access to NH-1,2,3-triazoles was possible using “alkyne-free” methods. Both, primary nitro compounds and aromatic aldehydes are easily accessible industrial scale products.^12^

One of the first denitrogenative transformations of 1,2,3-NH-triazoles 1 was reported in 2014. In this process, *in situ* sulfonylation with triflic anhydride and 2,6-di(*tert*-butyl)-4-methylpyridine (DTBMP) as a base was used to generate reactive *N*-triflyl triazoles 2.^13^ Their ring cleavage by a chiral Rh(ii) catalyst in the presence of an excess of alkene 3 afforded 2,3-dihydropyrroles 4 with low to good enantiocontrol (Scheme 3).

**Scheme 3:**
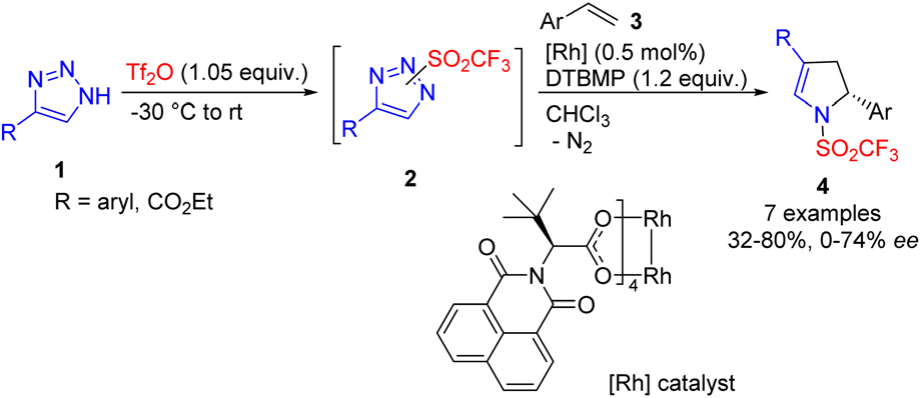
Synthesis of 2,3-dihydropyrroles by cleavage of NH-triazole in the presence of triflic anhydride. DTBMP – 2,6-di(*tert*-butyl)-4-methylpyridine.

However, besides triflation, there are no other examples of *in situ* sulfonylation of NH-1,2,3-triazoles followed by denitrogenative transformations. Therefore, this reaction is limited to the extremely electron-accepting triflyl group and analogous ring cleavage did not proceed with other *N*-sulfonyl triazoles.

In contrast to sulfonylation, acylation of NH-1,2,3-triazoles is more versatile and has developed into a highly active area of research in recent years.^14^ Tandem acylation followed by ring cleavage without isolation of *N*-acyltriazoles was performed using acyl halides or acid anhydrides. The mechanism of this transformation, recently confirmed by us,^14^ involved the formation of N1 (5) and N2-acylated (6) triazoles in equilibrium, followed by acid-mediated cleavage of the former. Denitrogenation and formation of a vinyl cation in an irreversible step was the driving force of N2–N1-acyltriazole interconversion, which ensured the complete transformation of triazoles into ring cleavage products 7–9 (Scheme 4).

**Scheme 4:**
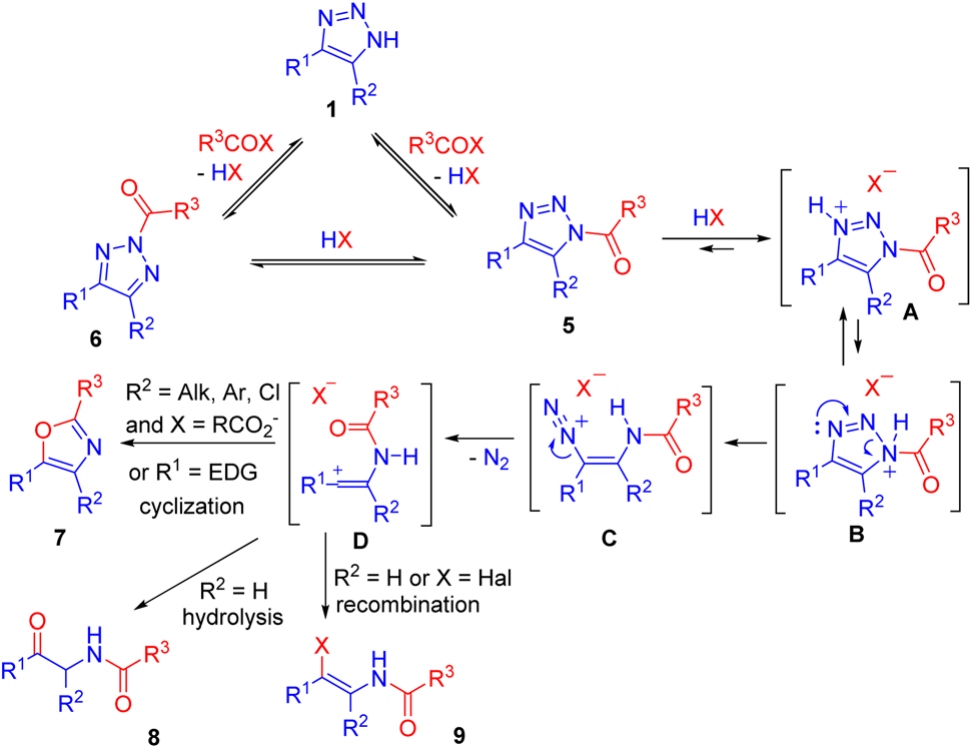
Mechanism of NH-triazole cleavage with electrophiles.

Cleavage of NH-1,2,3-triazoles 1 with an excess of acyl halides 10 (X = Cl, Br) under elevated temperature led to the formation of β-enamido halides 9 in moderate to good yields (Scheme 5).^15^ β-Enamido halides are difficult to access by other synthetic routes and are present in natural products, which underlines the synthetic value of the method.

**Scheme 5:**
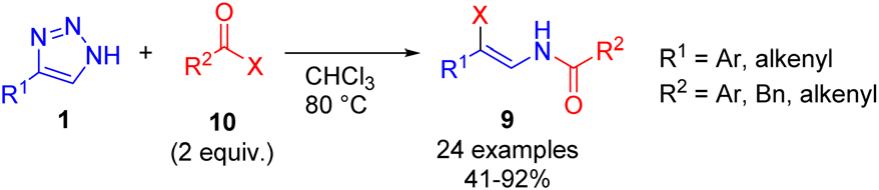
Cleavage of NH-1,2,3-triazoles with acyl halides.

This transformation in the presence of sodium sulfonates was employed in the synthesis of enamido triflates or sulfonates 11. Mainly compounds with the phenacyl group at the nitrogen were accessed by the mentioned route (Scheme 6).^15^

**Scheme 6:**
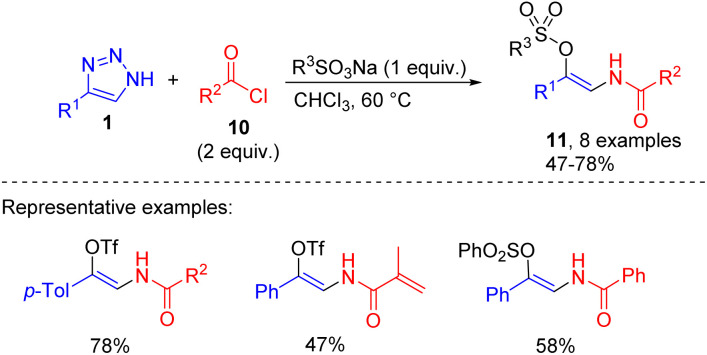
Synthesis of β-enamido triflates and sulfonates from NH-1,2,3-triazoles, acyl halides, and sodium sulfonates.

An alternative method, applicable to the synthesis of β-fluoroacylenamido triflates is based on the formation of N2-acyltriazoles 6*via* the *in situ* acylation of NH-1,2,3-triazoles with fluorinated acid anhydrides followed by their treatment with triflic acid, which proceeds through N2–N1 acyltriazole interconversion and ring cleavage (Scheme 7).^14^

**Scheme 7:**
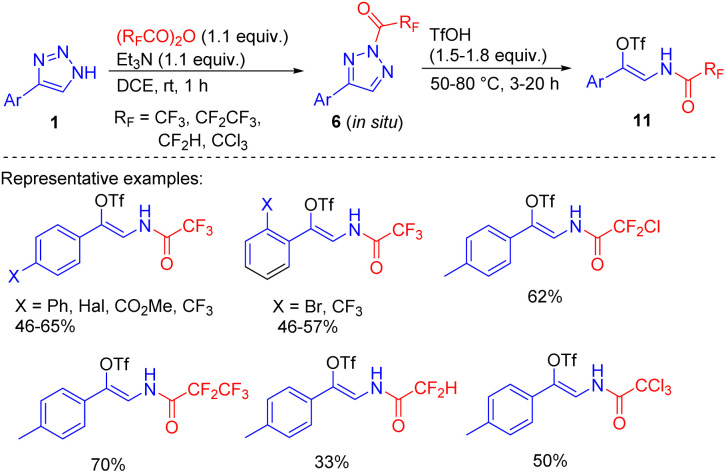
One-pot two step synthesis of β-fluoroacylenamido triflates. DCE = 1,2-dichloroethane.

The products are useful building blocks, that can get involved in Pd-catalysed cross-coupling substitution reactions of the triflate group to access multifunctionalized enamide derivatives – attractive drug candidates and synthetic intermediates.^16^ None of the methods mentioned was applicable with the less reactive alkyl-substituted acylating agents (Ac_2_O, AcCl), because the resulting acyltriazoles were resistant to ring cleavage even at elevated temperatures.^14^

The cleavage of NH-1,2,3-triazoles with an excess of fluoroalkylated acid anhydrides led to highly pharmaceutically relevant 2-fluoroalkyl oxazoles 7 (in the cases of 4,5-disubstituted triazoles) or 2-acylaminoketones (for 4-substituted triazoles, R = H) (Scheme 8).^17^ In the first case intramolecular cyclization took place, whereas in the second, unstable β-acyloxyenamide 8′ formed, which underwent ester hydrolysis to 2-acylaminoketone 8 upon treatment with an aqueous base. The difference in chemoselectivity was attributed to the increased vinyl cation stability of disubstituted examples, which made them more prone to intramolecular cyclization.

**Scheme 8:**
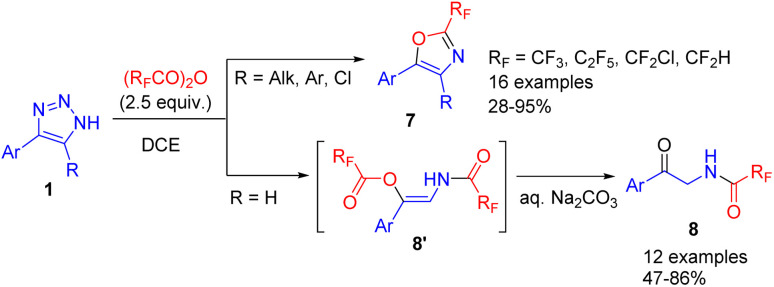
Synthesis of fluoroalkylated oxazoles and 2-acylaminoketones from NH-1,2,3-triazoles with fluoroalkylated acid anhydrides.

4,5-disubstituted NH-1,2,3-triazole reacted with trichloroacetic anhydride to give 2-unsubstituted oxazole 13, due to the low stability of the trichloromethyl-substituted product 12 during silica gel column chromatography. The whole transformation is a rare and unique case of a reaction involving trichloroacetic anhydride as a one-carbon building block (Scheme 9).^17^

**Scheme 9:**
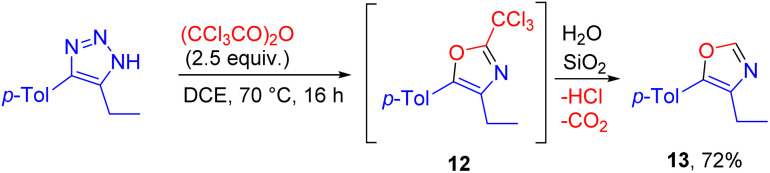
Formation of 2-unsubstituted oxazole from NH-1,2,3-triazole and trichloroacetic anhydride.

The limitation of oxazole synthesis to only disubstituted triazoles was overcome by the cyclization of *in situ* formed β-acyloxyenamide 8′ to oxazoles 7 using Et_3_N and proceeded quickly and nearly quantitatively under ambient conditions.^18^ This one-pot triazole cleavage procedure provided an efficient access to 2-fluoroalkylated oxazoles from monosubstituted triazoles in good to excellent yields (Scheme 10).

**Scheme 10:**
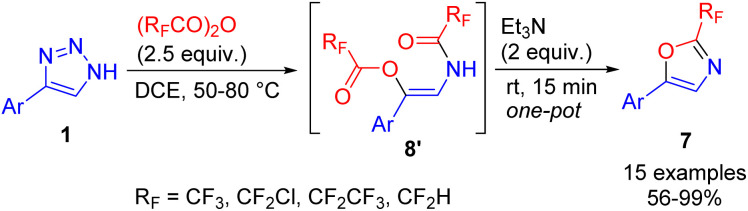
One-pot synthesis of fluoroalkylated oxazoles from NH-1,2,3-triazoles, acid anhydrides and Et_3_N.

The easy access to fluorinated 2-acylaminoketones 8 was utilized in a number of one-pot syntheses of fluoroalkylated heterocycles directly from NH-1,2,3-triazoles 1. First, 2-fluoroalkyl imidazoles 14 were prepared by cleavage with trifluoroacetic or perfluoropropanoic anhydrides, followed by the treatment of the ketamide intermediate with an aqueous solution of the primary amine (or ammonium acetate for R = H) under microwave conditions. The acid formed after hydrolysis of the enamide to yield 2-acylaminoketone promoted the Robinson–Gabriel cyclization of the latter. This procedure afforded imidazoles 14 in moderate to good yields in a one-pot manner starting from triazoles (Scheme 11).^17^

**Scheme 11:**
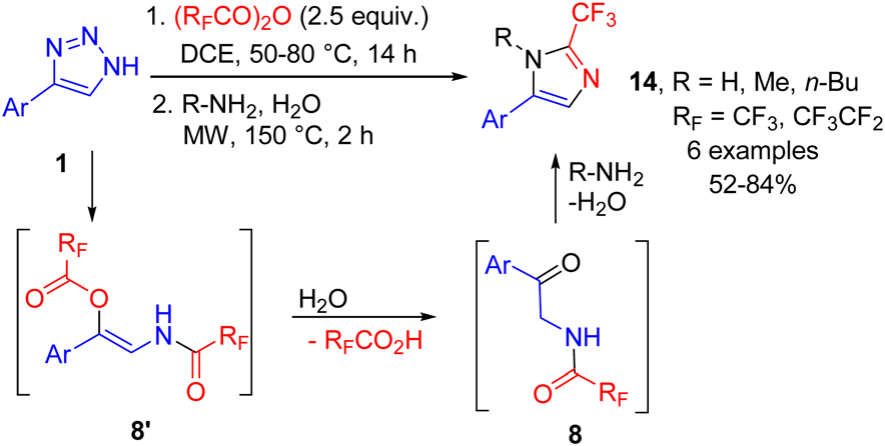
One-pot synthesis of 2-fluoroalkyl-imidazoles from NH-1,2,3-triazoles.

Alternatively, condensation of the formed acyloxyenamide 8′ with hydrazine hydrate after switching the solvent to acetic acid provided fluoroalkylated 1,2,4-triazines 15 (Scheme 12).^17^

**Scheme 12:**
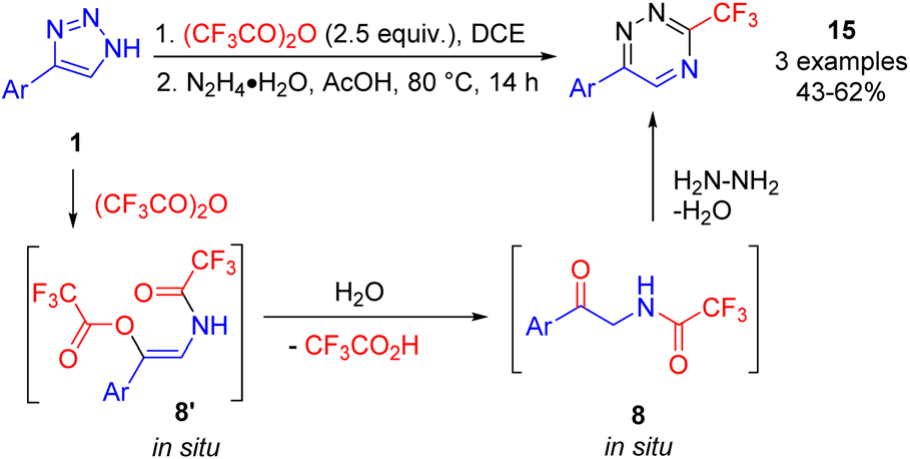
Synthesis of 3-fluoroalkyl-1,2,4-triazines from NH-1,2,3-triazoles.

The formation of the vinyl cation intermediate in *N*-acyltriazole cleavage was confirmed by changing the solvent from a chlorinated one to acetonitrile or propionitrile.^18^ In one special case (Scheme 13, R = *p*-Tol) adducts 16 of the Ritter reaction were formed and hydrolysed to bis(enamides) 17. However, this reaction was not general and in the cases of electron-richer triazoles, cyclization of the vinyl cation to oxazoles 7 took place (Scheme 13, R = H, EDG). This route is an alternative to one mentioned above (Scheme 10), and is applicable to electron-rich substrates. The straightforward formation of oxazoles 7 rather than enamides 8′ in polar MeCN was explained by the decreased stability of the vinyl cation–trifluoroacetate anion contact ion pair, which prevented recombination and favoured cyclization.^18^

**Scheme 13:**
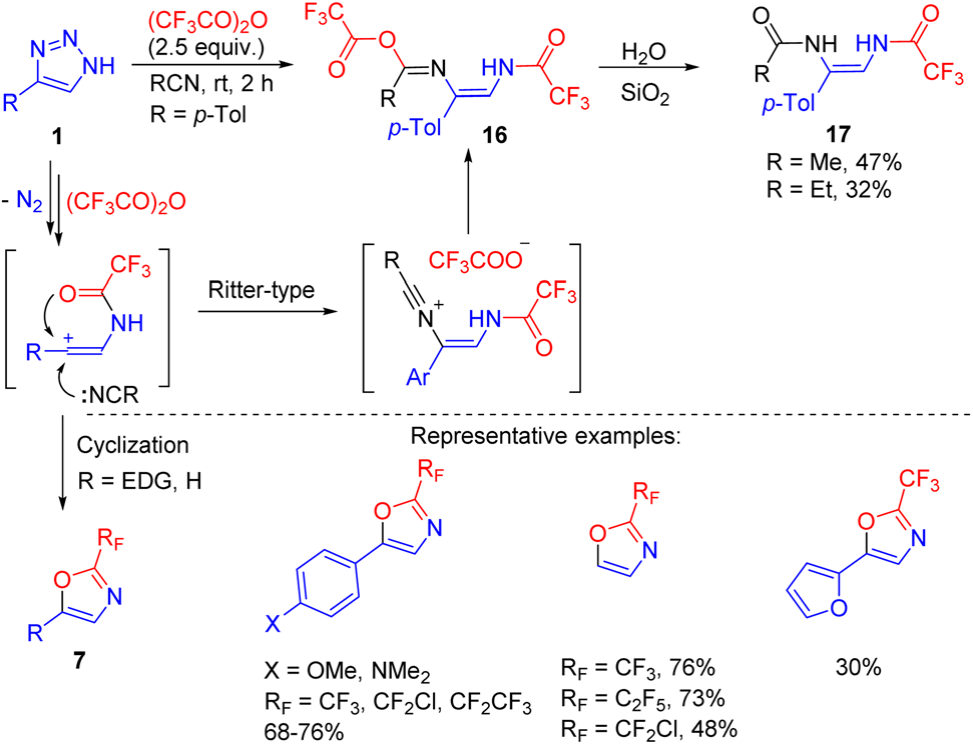
Formation of bis(enamides) 17 and oxazoles 7 by the cleavage of NH-1,2,3-triazoles with trifluoroacetic anhydride in nitrile solvent.

Several efficient NH-1,2,3-triazole ring cleavage protocols were also developed for NH-benzotriazole 18, which can be easily and regioselectively acylated on N1. The treatment of the formed *N*-acylbenzotriazole 20 with AlCl_3_ as a Lewis acid promotor facilitated ring cleavage leading to benzoxazoles under relatively harsh conditions (Scheme 14).^19^

**Scheme 14:**
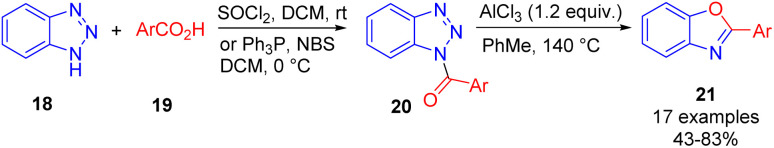
Synthesis of benzoxazoles from NH-benzotriazoles.

Rare examples of *ortho*-iodoacetanilide 22 formation in moderate yields from NH- and related *N*-acylbenzotriazole were reported in which the All_3_/Ac_2_O system or aluminium and iodine in acetonitrile were used.^20^ These are the only cases of *N*-acetylbenzotriazole 23 ring cleavage known. Importantly, the reaction of *N*-acetylbenzotriazole 23 with AlCl_3_ was not efficient and led only to deacylation, and not to the desired ring cleavage product (Scheme 15).^19^

**Scheme 15:**
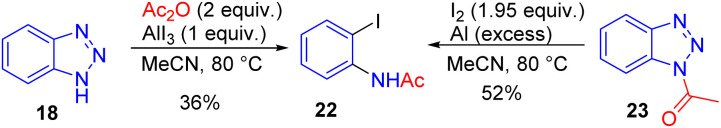
Formation of *o*-iodoacetanilide by AlI_3_-mediated cleavage of N1-acetylbenzotriazole.

Cleavage of electron-rich 4-aryl-NH-1,2,3-triazoles 1 was successfully achieved with thiophosgene leading to the formation of vinyl isothiocyanates 24 by HCl elimination from the vinyl chloride intermediate (Scheme 16).^21^ The vinyl isothiocyanate moiety is present in natural products with antifungal and antibacterial activity and is difficult to access by traditional methods. Switching from electron-rich aromatic NH-triazoles to unsubstituted NH-1,2,3-triazole afforded product 25 of HCl addition across the double bond in moderate yield.

**Scheme 16:**
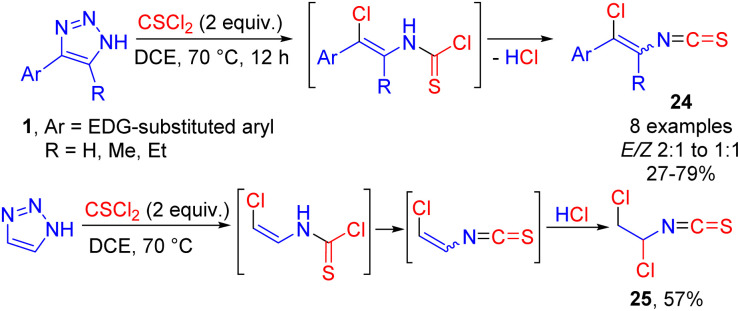
Cleavage of NH-1,2,3-triazoles with thiophosgene.

A similar transformation with triazoles 1 bearing an electron-rich aryl or alkenyl substituent in position 4 proceeded with triphosgene.^21^ The *in situ* formed carbamoyl chlorides 26 were treated with nucleophiles to gain access to multifunctional compounds 27, such as *N*-alkenyl carbamates, ureas and thiocarbamates (Scheme 17).

**Scheme 17:**
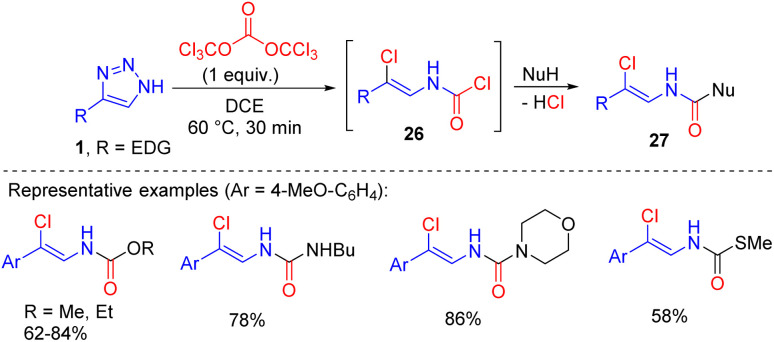
Synthesis of multifunctional *N*-alkenyl compounds by the cleavage of NH-1,2,3-triazoles with triphosgene.

The denitrogenative transformation of NH-1,2,3-triazoles was studied also on more complex substrates such as 4-(1-hydroxycyclobutyl)-1,2,3-triazoles 28. Their cleavage with acyl chlorides 10 catalysed by triflic acid provided efficient access to cyclic enaminones 29 (Scheme 18).^22^ The reaction proceeded *via* the cleavage of *N*-acyltriazole and semipinacol rearrangement cascade. The procedure was found to be easily scalable to give multifunctional substrates in good yields.

**Scheme 18:**
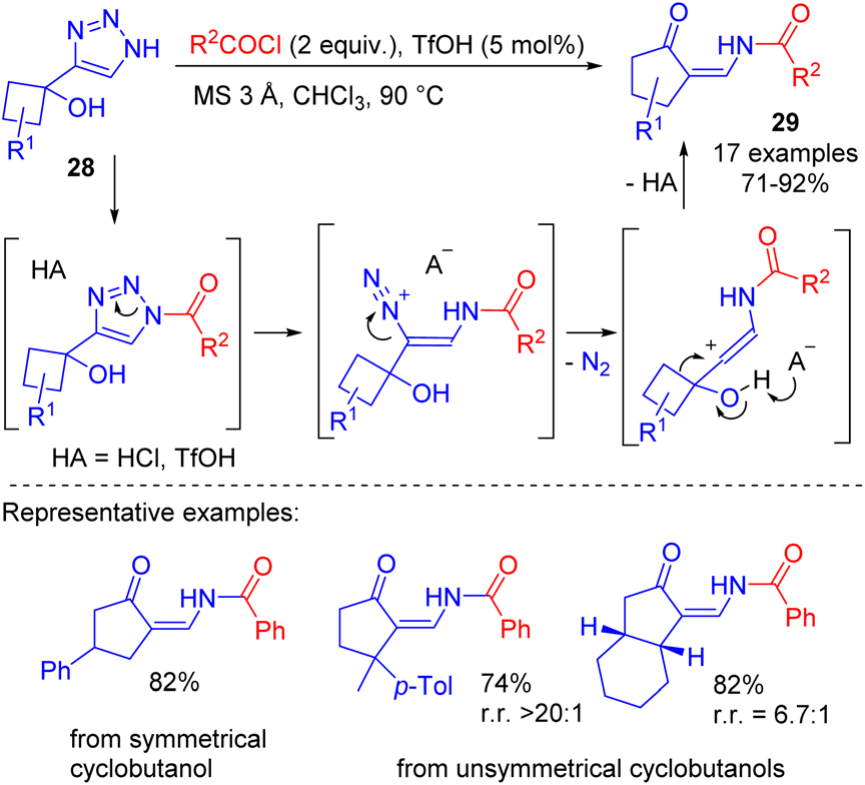
Synthesis of cyclic enaminones by TfOH-catalyzed cleavage of 4-(1-hydroxycyclobutyl)-1,2,3-triazoles with acyl chlorides.

## Conclusions

In conclusion, NH-1,2,3-triazoles are commercially available or easily synthesized starting materials that exhibit a remarkable versatility in transformations to diverse nitrogen-containing heterocycles and functionalized *N*-alkenyl compounds *via* denitrogenative cleavage. *In situ* prepared *N*-acylated 1,2,3-triazoles are key intermediates in these transformations. Acid-mediated triazole ring opening of *N*-acylated 1,2,3-triazoles, followed by nitrogen elimination affords vinyl cation intermediates, which undergo a variety of reactions such as cyclization or heteroatom capture. Further development of denitrogenation of NH-1,2,3-triazoles accompanied by C–C bond forming reactions, C–H insertion or rearrangement of the vinyl cation can be expected, providing access to a structural diversity of products with potential applications in drug development. Moreover, due to easy availability of NH-1,2,3-triazoles they are excellent starting materials for the development of new industrial synthetic processes.

## Conflicts of interest

There are no conflicts to declare.

## Supplementary Material

## References

[cit1] (a) Chemistry of 1,2,3-triazoles, in Topics in Heterocyclic Chemistry, ed. W. Dehaen and V. A. Bakulev, Springer, 2015, vol. 40

[cit2] Kumar S., Sharma B., Mehra V., Kumar V. (2021). Recent accomplishments on the synthetic/biological facets of pharmacologically active 1*H*-1,2,3-triazoles. Eur. J. Med. Chem..

[cit3] Tornøe C. W., Christensen C., Meldal M. (2002). Peptidotriazoles on Solid Phase: [1,2,3]-Triazoles by Regiospecific Copper(I)-Catalyzed 1,3-Dipolar Cycloadditions of Terminal Alkynes to Azides. J. Org. Chem..

[cit4] Akter M., Rupa K., Anbarasan P. (2022). 1,2,3-Triazole and Its Analogues: New Surrogates for Diazo Compounds. Chem. Rev..

[cit5] Motornov V., Markos A., Beier P. (2018). A rhodium-catalyzed transannulation of *N*-(per)fluoroalkyl-1,2,3-triazoles under microwave conditions – a general route to *N*-(per)fluoroalkyl-substituted five-membered heterocycles. Chem. Commun..

[cit6] Opsomer T., Dehaen W. (2021). Metal-free syntheses of *N*-functionalized and NH-1,2,3-triazoles: an update on recent developments. Chem. Commun..

[cit7] Prakash R., Opsomer T., Dehaen W. (2021). Triazolization of Enolizable Ketones with Primary Amines: A General Strategy toward Multifunctional 1,2,3-Triazoles. Chem. Rec..

[cit8] Jin T., Kamijo S., Yamamoto Y. (2004). Copper-Catalyzed Synthesis of *N*-Unsubstituted 1,2,3-Triazoles from Nonactivated Terminal Alkynes. Eur. J. Org Chem..

[cit9] Hui R., Zhao M., Chen M., Ren Z., Guan Z. (2017). One-Pot Synthesis of 4-Aryl-NH-1,2,3-Triazoles through Three-Component Reaction of Aldehydes, Nitroalkanes and NaN_3_. Chin. J. Chem..

[cit10] Jankovic D., Virant M., Gazvoda M. (2022). Copper-Catalyzed Azide–Alkyne Cycloaddition of Hydrazoic Acid Formed *In Situ* from Sodium Azide Affords 4-Monosubstituted-1,2,3-Triazoles. J. Org. Chem..

[cit11] Hu L., Muck-Lichtenfield C., Wang T., He G., Gao M., Zhao J. (2016). Reaction between Azidyl Radicals and Alkynes: A Straightforward Approach to NH-1,2,3-Triazoles. Chem. – Eur. J..

[cit12] Benzaldehydes are by the order of magnitude less expensive than corresponding aryl acetylenes according to price lists of common chemical suppliers. Also, nitroalkanes are very inexpensive large scale industrial products. See: Ono N., The Nitro Group in Organic Synthesis, John Wiley & Sons, New York, 2002

[cit13] Kwok S. W., Zhang L., Grimster N. P., Fokin V. V. (2014). Catalytic Asymmetric Transannulation of NH-1,2,3-Triazoles with Olefins. Angew. Chem., Int. Ed..

[cit14] Motornov V., Pohl R., Klepetářová B., Beier P. (2023). *N*-Acyl-1,2,3-triazoles – key intermediates in denitrogenative transformations. Chem. Commun..

[cit15] Wang T., Tang Z., Luo H., Tian Y., Xu M., Lu Q., Li B. (2021). Access to (*Z*)-β-Substituted Enamides from *N*1-H-1,2,3-Triazoles. Org. Lett..

[cit16] Lu Q., Wang T., Wu Q., Cheng L., Luo H., Liu L., Chu G., Wang L., Li B. (2022). C–H heteroarylation of aromatics *via* catalyst free S_N_2′ coupling cycloaromatization. Green Chem..

[cit17] Motornov V., Beier P. (2022). Access to Fluoroalkylated Azoles and 2-Acylaminoketones *via* Fluorinated Anhydride-Mediated Cleavage of NH-1,2,3-Triazoles. Org. Lett..

[cit18] Motornov V., Beier P. (2022). One-pot synthesis of 4-substituted 2-fluoroalkyloxazoles from NH-1,2,3-triazoles and fluoroalkylated acid anhydrides. New J. Chem..

[cit19] Singh A. S., Mishra N., Kumar D., Tiwari V. K. (2017). Lewis-Acid-Mediated Benzotriazole Ring Cleavage (BtRC) Strategy for the Synthesis of 2-Aryl Benzoxazoles from *N*-Acylbenzotriazoles. ACS Omega.

[cit20] Dong B., Liu Y., Yang P., Sang D., Tian J., Li L., Long S. (2022). Denitrogenative cleavage of benzotriazoles and benzotriazinones, and selective *N*-desulfonylation of benzotriazoles by aluminum halides. Tetrahedron Lett..

[cit21] Motornov V., Beier P. (2023). Synthesis of *N*-vinyl isothiocyanates and carbamates by the cleavage of NH-1,2,3-triazoles with one-carbon electrophiles. Org. Biomol. Chem..

[cit22] Xu M., Liu L., Wang T., Luo H., Hou M., Du L., Xin X., Lu Q., Li B. (2022). Acid-catalyzed ring-expansion of 4-(1-hydroxycyclobutyl)-1,2,3-triazoles. Org. Chem. Front..

